# Correction to: Coronaviruses: a challenge of today and a call for extended human postmortem brain analyses

**DOI:** 10.1007/s00702-021-02356-6

**Published:** 2021-06-19

**Authors:** Peter Riederer, Volker ter Meulen

**Affiliations:** 1 Clinic and Policlinic for Psychiatry, Psychosomatics and Psychotherapy, University Hospital Würzburg, Margarete-Hoeppel-Platz 1, 97080 Würzburg, Germany; 2 University of Southern Denmark Odense, J.B. Winslows Vey 18, 5000 Odense, Denmark; 3 Institut für Virologie und Immunbiologie, Universität Würzburg, Versbacherstraße Straße 7, 97078 Würzburg, Germany

## Correction to: Journal of Neural Transmission (2020) 127(9):1217–1228 10.1007/s00702-020-02230-x

The article Coronaviruses: a challenge of today and a call for extended human postmortem brain analyses, written by Peter Riederer and Volker ter Meulen, was originally published Online First without Open Access. After publication in volume 127, issue 9, page 1217–1228 the author decided to opt for Open Choice and to make the article an Open Access publication. Therefore, the copyright of the article has been changed to © The Author(s) 2021 and this article is licensed under a Creative Commons Attribution 4.0 International License, which permits use, sharing, adaptation, distribution and reproduction in any medium or format, as long as you give appropriate credit to the original author(s) and the source, provide a link to the Creative Commons licence, and indicate if changes were made. The images or other third party material in this article are included in the article’s Creative Commons licence, unless indicated otherwise in a credit line to the material. If material is not included in the article’s Creative Commons licence and your intended use is not permitted by statutory regulation or exceeds the permitted use, you will need to obtain permission directly from the copyright holder. To view a copy of this licence, visit http://creativecommons.org/licenses/by/4.0/.

The original article has been corrected.

